# Report of a Case of Epidemic Cerebro-Spinal Meningitis

**Published:** 1899-09

**Authors:** W. T. Howard

**Affiliations:** Cleveland; Professor of Pathology, Western Reserve University; Pathologist to the Lakeside and City Hospitals


### Abstracts and Selections.

### REPORT OF A CASE OF EPIDEMIC CEREBRO-SPINAL MENINGITIS.

### With Exhibition of Cultures of the Diplococcus Intracellularis Meningitidis Obtained from the Cerebro-spinal Exudation.*

BY W. T. HOWARD, JR., M. D., CLEVELAND.
PROFESSOR OF PATHOLOGY, WESTERN RESERVE UNIVERSITY; PATHOLOGIST TO THE LAKESIDE AND CITY HOSPITALS.

(FROM THE PATHOLOGIC LABORATORY OF LAKESIDE HOSPITAL.)

I wish to show this evening cultures of the *diplococcus intracellularis meningitidis,* obtained from the cerebro-spinal exudation of a case of epidemic cerebro-spinal meningitis. As you see, upon the surface of the blood serum there is a delicate growth of fine, round, whitish, shining colonies with rather regular outlines. In some places the colonies are scattered, while in others they are confluent. Coverslips made from the cultures obtained in this case show diplococci rather smaller than the ordinary micrococci. They are often not quite round. They lack the lancet-shape of the *diplococcus lanceolatus* and resemble in shape the gonococcus more than any other known organism.

Coverslips made at the necropsy from the cerebro-spinal exudation showed numbers of diplococci within polymorphonuclear leucocytes. They were never found free. The diplococci found in the exudation correspond exactly in appearance with those growing in the cultures. Beside these a few scattered short bacilli were seen in our preparation. The diplococcus found in this case grows poorly on agar and glycerine-agar, on which it forms flat, fine, white, translucent colonies.

There was a feeble growth in bouillon, which was rendered slightly cloudy, giving rise to viscid cloudiness on shaking. In this medium short chains of four or even six cocci were sometimes seen. This organism has distinct culture characteristics which serve to distinguish it from the other pathogenic micrococci.

It was first described in 1887 by Weichselbaum of Vienna, who

*Read before the Cleveland Medical Society.

found it in six cases of cerebro-spinal meningitis. On account of its nearly constant presence in the pus-cells in the exudate, he called it the *diplococcus intracellularis meningitidis*. Later the organism was found in cases of meningitis by Goldsmith and Netter. In 1895 Jäger found it in twelve cases of epidemic cerebro-spinal meningitis occurring in Stuttgardt. Since then Heubner, Holdheim, Peterson, Fürbringer, Kischensky, Kister, Stveltzner, Finklestein, Sherer and Councilman, with Mallory and Wright and Livingood have found this organism in cerebro-spinal meningitis either at necropsy or in fluid obtained by lumbar puncture.

Councilman's contributions are the latest and most valuable. He, with Mallory and Wright, studied 111 cases in the Boston epidemic of 1896-7; thirty-five of these cases came to necropsy, and of these the diplococcus was found in all but two cases. Lumbar puncture was performed in 50 cases and the organism found in 38. On account of the difficulty with which the organism grows, Councilman advises that a number of cultures be made from the suspected material. In many cases in which the organism was found in the pus-cells, in the fresh exudate, and in hardened sections, cultures remained sterile.

I would recommend you to read this classic contribution of Councilman's, which was published in 1898 by the Massachusetts State Board of Health. It is of special interest to us in northern Ohio, as we are now having a mild epidemic of this form of cerebro-spinal meningitis. One of the many important and interesting points brought out by Councilman is that this is the least fatal variety of meningitis. According to Councilman in the various epidemics from twenty-five to seventy-five per cent. die. This is in striking contrast with what obtains in meningitis due to other bacteria. Of the 111 cases reported by Councilman the mortality was sixty-eight and one-half per cent. The case from which these cultures were obtained was a patient in Lakeside Hospital, in the service of my colleague, Dr. J. H. Lowman, to whose courtesy I am indebted for the clinical history. H. M., negro, aged 23 years, entered the hospital April 3, 1899, complaining of headache and general malaise. His family history is without interest. He had typhoid four years ago. He had measles in childhood and has had gonorrhœa twice. He denied syphilis. On admission he stated that he had not been feeling well for a week. On April 1, he had some headache. On admission in the evening his temperature was 102°. The next morning it was normal. The pulse was regular and rhythmic, and not accelerated. He was well developed and well nourished. Examination of the chest and abdomen was negative. The examination of the blood showed 27,366 leucocytes per cubic millimeter. The increase was in the polymorphonuclear neutrophilic cells. On April 5, the leucocytes were 19,-400. The chief symptoms were occipital headache and cervical opisthotonos. He was delirious at times. The special senses were not affected. He vomited once. On April 6 there was rigidity of the muscles of the back and slight frothing at the mouth. Death occurred in 15 minutes. The necropsy was begun eight hours after death, the body having been kept in the meantime in the refrigerator at 30° F. Anatomic diagnosis: Serous and purulent cerebro-spinal meningitis. There was general arterial hypoplasia with hypertrophy of the left ventricle of the heart and chronic passive congestion of the lungs, liver, spleen and kidneys. The *diplococcus intracellularis meningitidis* was found in the cerebro-spinal exudation. Colonies of an unidentified non-pathogenic bacillus occurred in the cultures from the cerebral meninges and the spleen.

The head was of ordinary size and shape. The scalp and skull were thick. The *dura mater* was congested, but otherwise negative. On opening the dura a large amount of cloudy serum escaped. The sinuses contained a large amount of dark red blood. The meninges were everywhere congested. There was no exudation from the cortical meninges. The surface of the cerebrum was negative. The meninges at the base of the brain, especially over the inferior surface of the cerebellum, the pons and medulla were the seat of a thin purulent exudation, which included the cranial nerves arising in this region. The optic and olfactory nerves were free. On section the cerebrum and cerebellum, pons and medulla were congested, otherwise negative. The lateral ventricles contained an excess of cloudy serum. The choroid plexuses were congested. The spinal meninges were congested, and there was a large amount of cloudy serous fluid, containing larger and smaller flakes of fibrin. The detailed report of the examination of the other organs is without interest here, and will therefore be omitted.

Histologic examination of the cerebral cortex, the cerebellum, the pons and the medulla stained in eosin and methylen-blue show, in the main, the same changes. The vessels of the meninges are dilated and markedly congested. In many there is no increase in leucocytes, while in others there are large numbers of polymorphonuclear neutrophiles and large mononuclear cells. The meningeal exudation varies in thickness and in amount. It is thickest at the inferior surface of the cerebellum and thinnest on the lateral surface of the cerebellum. The exudation consists of red blood corpuscles, polymorphonuclear neutrophiles, plasma-cells, large mononuclear leucocytes, and pia cells. The latter vary very much in size. Some are spindle-shaped, while others are oval or round. Many are very large, from twice to four times the size of polymorphonuclear leucocytes. Some of these contain two or three leucocytes. A small amount of fibrillated fibrin is seen. Following the course of many of the vessels in sulci of the cerebellum and cerebrum there is marked cellular exudation, similar to that described in the meninges. Some of the leucocytes and pia cells contain yellow pigment. Very few cocci can be found. Those are seen within polymorphonuclear leucocytes. Sections of the cord made at various levels fail to show any cellular exudation in the meninges or in the cord. The blood vessels are dilated. No special changes are made out in the nerve cells of the cord or brain.

Note.—Since the above case was reported, I have had the opportunity of studying two other cases. The first case is a girl of about 20 years of age, who has been ill about a month in the medical ward of Lakeside Hospital in the service of Dr. J. H. Lowman. A few days after admission 50 c. c. of slightly clouded serum, obtained from the case by lumbar puncture were submitted to me for examination. In the centrifugalized specimen there were a few polymorphonuclear leucocytes, a few of which contained large numbers of small diplococci. Blood serum cultures were made, some from the sediment and others by pouring several cubic centimeters of the cerebro-spinal fluid into the culture tubes, and all gave pure cultures of the *diplococcus intracellularis meningitidis*. This case is running a very chronic course.

For the second case I am indebted to the courtesy of Dr. Henry Wickes of the United States Marine Hospital Service. J. M., aged 28, entered the United States Marine Hospital May 31, 1899, complaining of pain in the back and neck. He has never had any other disease but measles. He came to Cleveland on an ore boat from Wisconsin, and came at once from the dock to the hospital. He said he had been ill two days. He developed cervical opisthotonos and coma and died June 4.

At the necropsy, which was held eighteen hours after death, there was well marked, purulent, basal meningitis. All the cranial nerves were surrounded by a purulent exudation. This was thickest on the inferior surface of the cerebellum. The spinal meninges contained a large amount of cloudy serum. The lateral ventricles contained a small amount of thick pus. There were scattered areas of broncho-pneumonia in the lower lobes of the lungs. The spleen was congested. The liver and kidneys and heart were fatty. Coverslip preparations from the cerebral and spinal exudation and from the pus from the lateral ventricles showed a few small diplococci inside polymorphonuclear leucocytes. No other bacteria were present. From the lungs capsulated diplococci were obtained. Blood serum cultures from all organs except the lungs remained sterile. From the pneumonic areas of the lungs a number of colonies of the *diplococcus lanceolatus* and a few of the *stapphylococcus pyogenes aureus* grew.—*Cleveland Journal of Medicine,* August, 1899.

